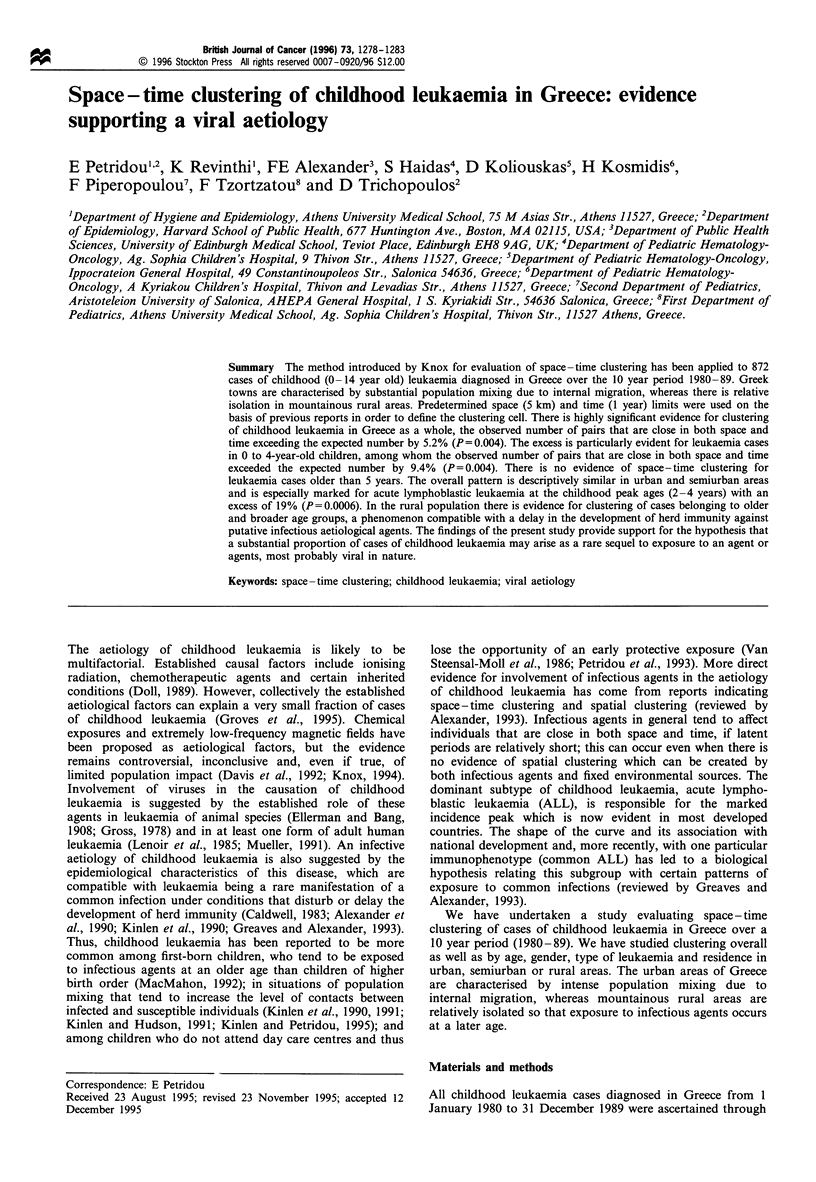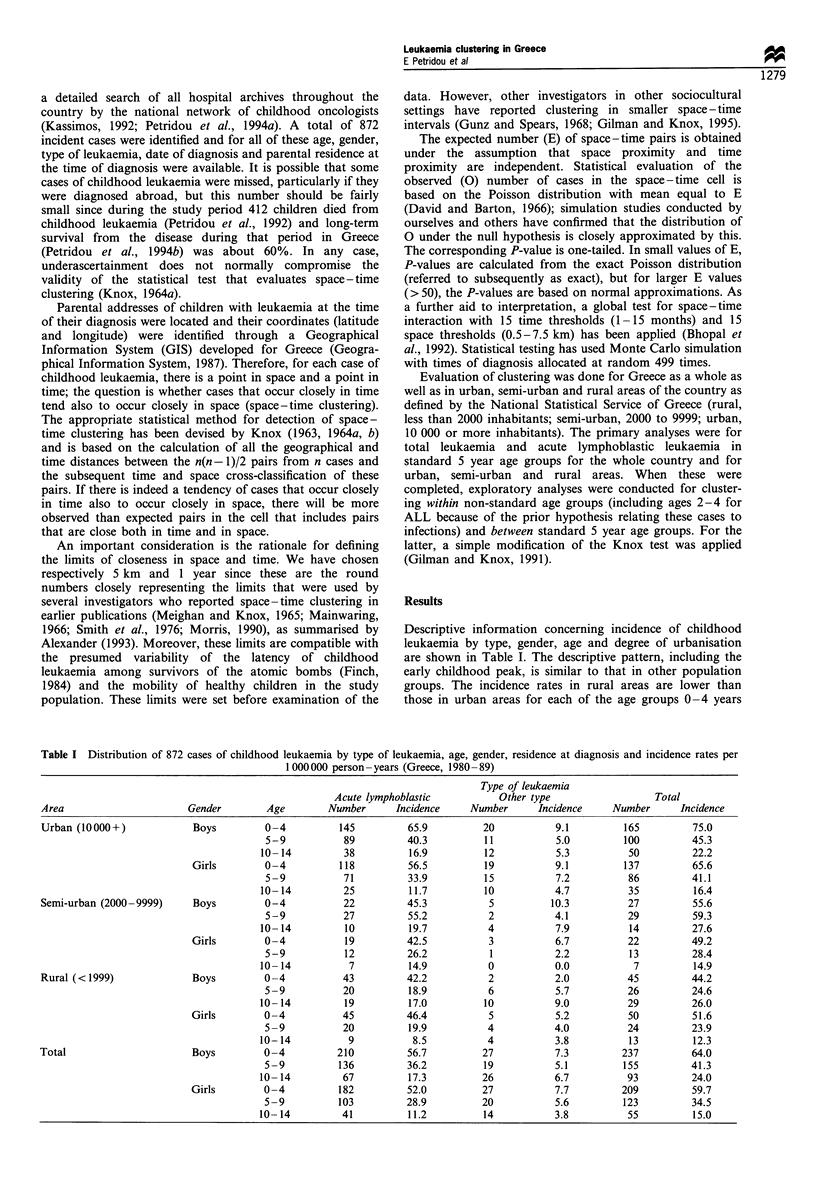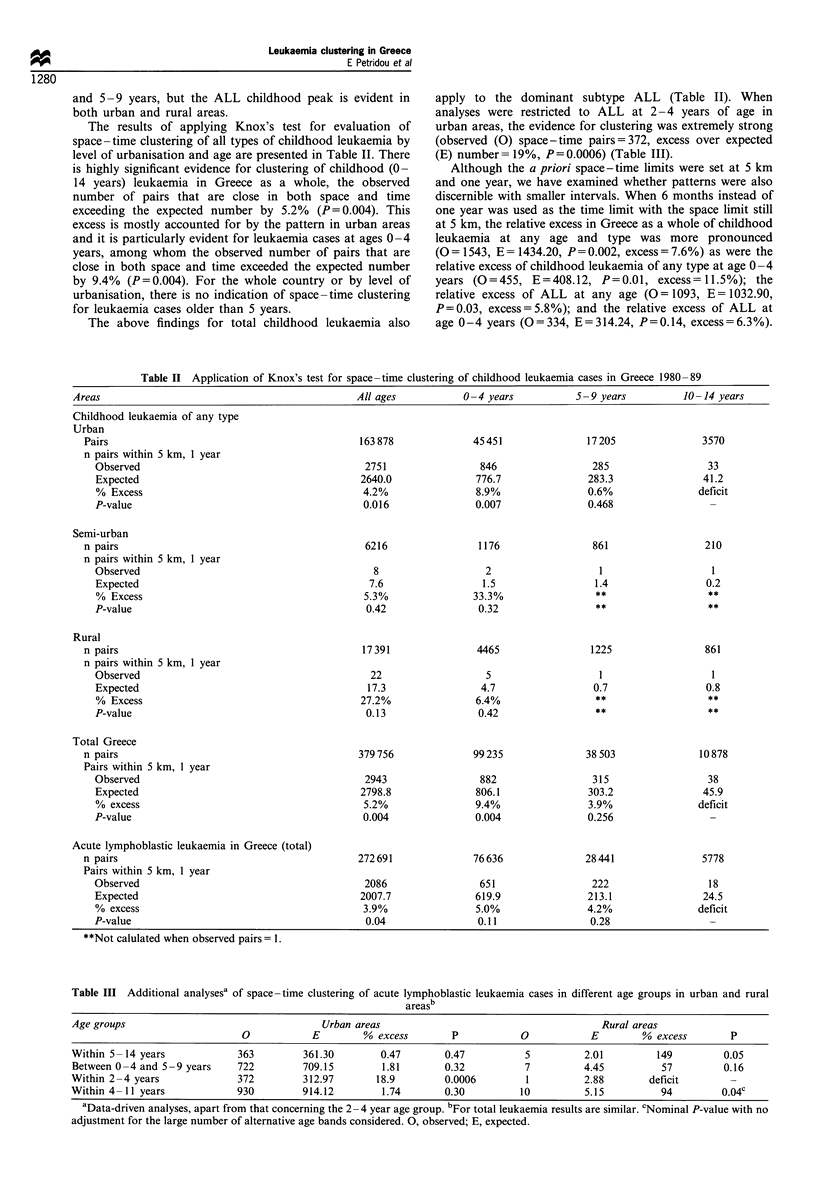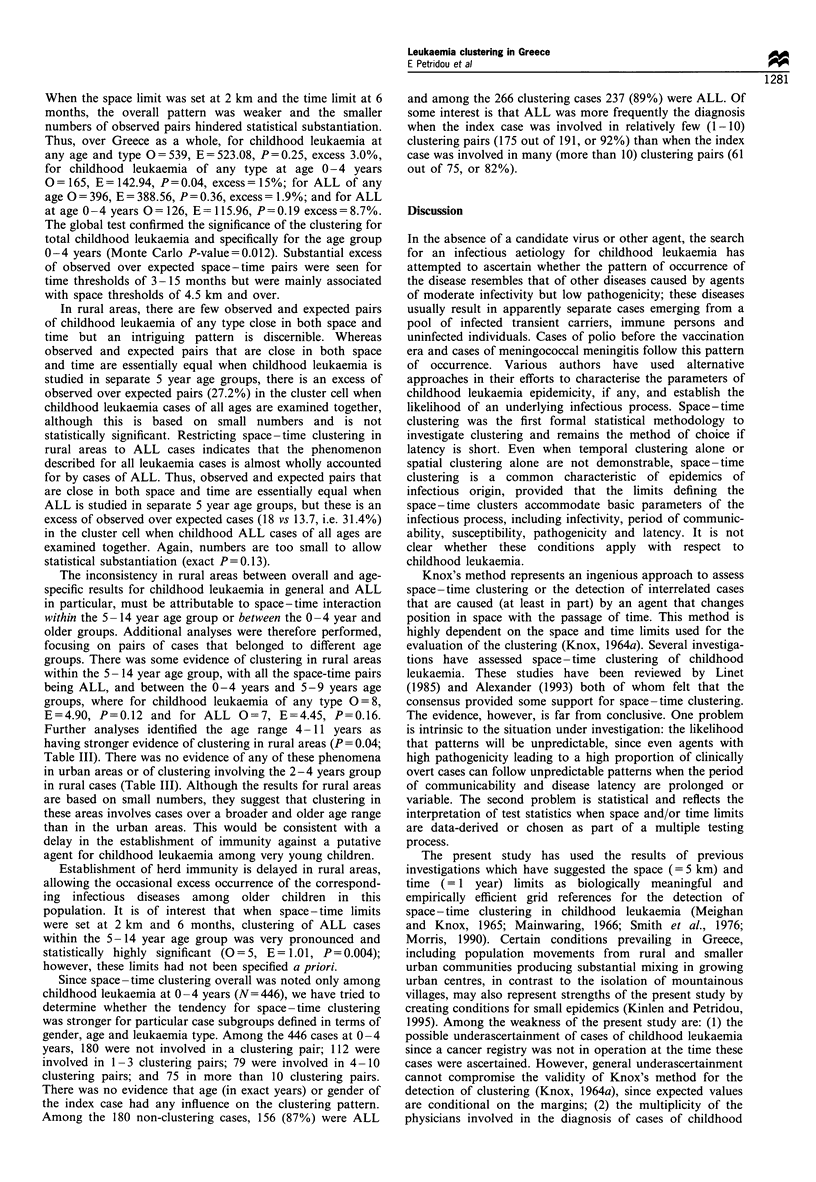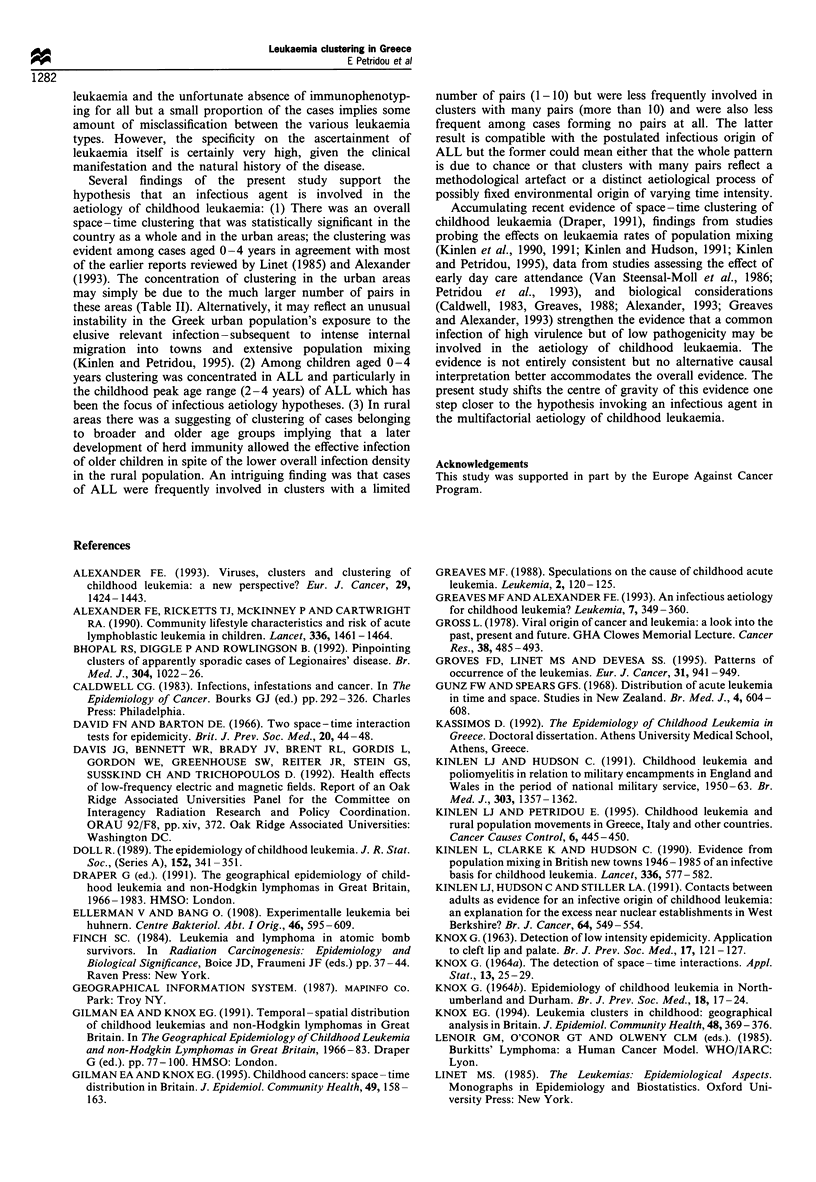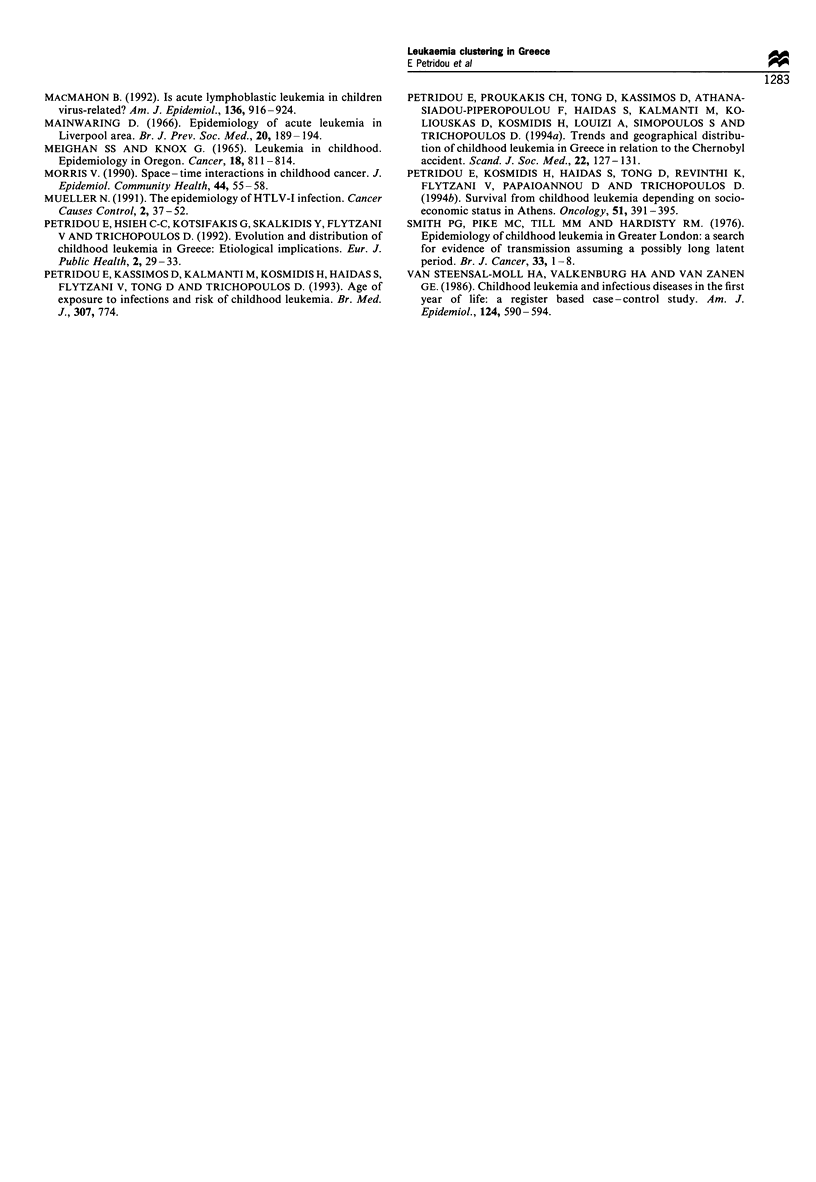# Space-time clustering of childhood leukaemia in Greece: evidence supporting a viral aetiology.

**DOI:** 10.1038/bjc.1996.245

**Published:** 1996-05

**Authors:** E. Petridou, K. Revinthi, F. E. Alexander, S. Haidas, D. Koliouskas, H. Kosmidis, F. Piperopoulou, F. Tzortzatou, D. Trichopoulos

**Affiliations:** Department of Hygiene and Epidemiology, Athens University Medical School, Greece.

## Abstract

The method introduced by Knox for evaluation of space-time clustering has been applied to 872 cases of childhood (0-14 year old) leukaemia diagnosed in Greece over the 10 year period 1980-89. Greek towns are characterised by substantial population mixing due to internal migration, whereas there is relative isolation in mountainous rural areas. Predetermined space (5 km) and time (1 year) limits were used on the basis of previous reports in order to define the clustering cell. There is highly significant evidence for clustering of childhood leukaemia in Greece as a whole, the observed number of pairs that are close in both spaces and time exceeding the expected number by 5.2% (P = 0.004). The excess is particularly evident for leukaemia cases in 0 to 4-year-old children, among whom the observed number of pairs that are close in both space and time exceeded the expected number by 9.4% (P = 0.004). There is no evidence of space-time clustering for leukaemia cases older than 5 years. The overall pattern is descriptively similar in urban and semiurban areas and is especially marked for acute lymphoblastic leukaemia at the childhood peak ages (2-4 years) with an excess of 19% (P = 0.0006). In the rural population there is evidence for clustering of cases belonging to older and broader age groups, a phenomenon compatible with a delay in the development of herd immunity against putative infectious aetiological agents. The findings of the present study provide support for the hypothesis that a substantial proportion of cases of childhood leukaemia may arise as a rare sequel to exposure to an agent or agents, most probably viral in nature.


British Journal of Cancer (1996) 73, 1278–1283
© 1996 Stockton Press All rights reserved 0007–0920/96 $12.00

### Space–time clustering of childhood leukaemia in Greece: evidence supporting a viral aetiology

E Petridou[1,2], K Revinthi[1], FE Alexander[3], S Haidas[4], D Koliouskas[5], H Kosmidis[6], F Piperopoulou[7], F Tzortzatou[8] and D Trichopoulos[2]

[1]Department of Hygiene and Epidemiology, Athens University Medical School, 75 M Asias Str., Athens 11527, Greece; [2]Department of Epidemiology, Harvard School of Public Health, 677 Huntington Ave., Boston, MA 02115, USA; [3]Department of Public Health Sciences, University of Edinburgh Medical School, Teviot Place, Edinburgh EH8 9AG, UK; [4]Department of Pediatric Hematology-Oncology, Ag. Sophia Children's Hospital, 9 Thivon Str., Athens 11527, Greece; [5]Department of Pediatric Hematology-Oncology, Ippocrateion General Hospital, 49 Constantinoupoleos Str., Salonica 54636, Greece; [6]Department of Pediatric Hematology-Oncology, A Kyriakou Children's Hospital, Thivon and Levadias Str., Athens 11527, Greece; [7]Second Department of Pediatrics, Aristoteleion University of Salonica, AHEPA General Hospital, 1 S. Kyriakidi Str., 54636 Salonica, Greece; [8]First Department of Pediatrics, Athens University Medical School, Ag. Sophia Children's Hospital, Thivon Str., 11527 Athens, Greece.

**Summary** The method introduced by Knox for evaluation of space–time clustering has been applied to 872 cases of childhood (0–14 year old) leukaemia diagnosed in Greece over the 10 year period 1980–89. Greek towns are characterised by substantial population mixing due to internal migration, whereas there is relative isolation in mountainous rural areas. Predetermined space (5 km) and time (1 year) limits were used on the basis of previous reports in order to define the clustering cell. There is highly significant evidence for clustering of childhood leukaemia in Greece as a whole, the observed number of pairs that are close in both space and time exceeding the expected number by 5.2% ($P=0.004$). The excess is particularly evident for leukaemia cases in 0 to 4-year-old children, among whom the observed number of pairs that are close in both space and time exceeded the expected number by 9.4% ($P=0.004$). There is no evidence of space–time clustering for leukaemia cases older than 5 years. The overall pattern is descriptively similar in urban and semiurban areas and is especially marked for acute lymphoblastic leukaemia at the childhood peak ages (2–4 years) with an excess of 19% ($P=0.0006$). In the rural population there is evidence for clustering of cases belonging to older and broader age groups, a phenomenon compatible with a delay in the development of herd immunity against putative infectious aetiological agents. The findings of the present study provide support for the hypothesis that a substantial proportion of cases of childhood leukaemia may arise as a rare sequel to exposure to an agent or agents, most probably viral in nature.

**Keywords:** space–time clustering; childhood leukaemia; viral aetiology

The aetiology of childhood leukaemia is likely to be multifactorial. Established causal factors include ionising radiation, chemotherapeutic agents and certain inherited conditions (Doll, 1989). However, collectively the established aetiological factors can explain a very small fraction of cases of childhood leukaemia (Groves *et al.*, 1995). Chemical exposures and extremely low-frequency magnetic fields have been proposed as aetiological factors, but the evidence remains controversial, inconclusive and, even if true, of limited population impact (Davis *et al.*, 1992; Knox, 1994). Involvement of viruses in the causation of childhood leukaemia is suggested by the established role of these agents in leukaemia of animal species (Ellerman and Bang, 1908; Gross, 1978) and in at least one form of adult human leukaemia (Lenoir *et al.*, 1985; Mueller, 1991). An infective aetiology of childhood leukaemia is also suggested by the epidemiological characteristics of this disease, which are compatible with leukaemia being a rare manifestation of a common infection under conditions that disturb or delay the development of herd immunity (Caldwell, 1983; Alexander *et al.*, 1990; Kinlen *et al.*, 1990; Greaves and Alexander, 1993). Thus, childhood leukaemia has been reported to be more common among first-born children, who tend to be exposed to infectious agents at an older age than children of higher birth order (MacMahon, 1992); in situations of population mixing that tend to increase the level of contacts between infected and susceptible individuals (Kinlen *et al.*, 1990, 1991; Kinlen and Hudson, 1991; Kinlen and Petridou, 1995); and among children who do not attend day care centres and thus lose the opportunity of an early protective exposure (Van Steensal-Moll *et al.*, 1986; Petridou *et al.*, 1993). More direct evidence for involvement of infectious agents in the aetiology of childhood leukaemia has come from reports indicating space–time clustering and spatial clustering (reviewed by Alexander, 1993). Infectious agents in general tend to affect individuals that are close in both space and time, if latent periods are relatively short; this can occur even when there is no evidence of spatial clustering which can be created by both infectious agents and fixed environmental sources. The dominant subtype of childhood leukaemia, acute lymphoblastic leukaemia (ALL), is responsible for the marked incidence peak which is now evident in most developed countries. The shape of the curve and its association with national development and, more recently, with one particular immunophenotype (common ALL) has led to a biological hypothesis relating this subgroup with certain patterns of exposure to common infections (reviewed by Greaves and Alexander, 1993).

We have undertaken a study evaluating space–time clustering of cases of childhood leukaemia in Greece over a 10 year period (1980–89). We have studied clustering overall as well as by age, gender, type of leukaemia and residence in urban, semiurban or rural areas. The urban areas of Greece are characterised by intense population mixing due to internal migration, whereas mountainous rural areas are relatively isolated so that exposure to infectious agents occurs at a later age.

### Materials and methods

All childhood leukaemia cases diagnosed in Greece from 1 January 1980 to 31 December 1989 were ascertained through

Correspondence: E Petridou
Received 23 August 1995; revised 23 November 1995; accepted 12 December 1995

a detailed search of all hospital archives throughout the country by the national network of childhood oncologists (Kassimos, 1992; Petridou *et al.*, 1994*a*). A total of 872 incident cases were identified and for all of these age, gender, type of leukaemia, date of diagnosis and parental residence at the time of diagnosis were available. It is possible that some cases of childhood leukaemia were missed, particularly if they were diagnosed abroad, but this number should be fairly small since during the study period 412 children died from childhood leukaemia (Petridou *et al.*, 1992) and long-term survival from the disease during that period in Greece (Petridou *et al.*, 1994*b*) was about 60%. In any case, underascertainment does not normally compromise the validity of the statistical test that evaluates space–time clustering (Knox, 1964*a*).

Parental addresses of children with leukaemia at the time of their diagnosis were located and their coordinates (latitude and longitude) were identified through a Geographical Information System (GIS) developed for Greece (Geographical Information System, 1987). Therefore, for each case of childhood leukaemia, there is a point in space and a point in time; the question is whether cases that occur closely in time tend also to occur closely in space (space–time clustering). The appropriate statistical method for detection of space–time clustering has been devised by Knox (1963, 1964*a*, *b*) and is based on the calculation of all the geographical and time distances between the $n(n-1)/2$ pairs from $n$ cases and the subsequent time and space cross-classification of these pairs. If there is indeed a tendency of cases that occur closely in time also to occur closely in space, there will be more observed than expected pairs in the cell that includes pairs that are close both in time and in space.

An important consideration is the rationale for defining the limits of closeness in space and time. We have chosen respectively 5 km and 1 year since these are the round numbers closely representing the limits that were used by several investigators who reported space–time clustering in earlier publications (Meighan and Knox, 1965; Mainwaring, 1966; Smith *et al.*, 1976; Morris, 1990), as summarised by Alexander (1993). Moreover, these limits are compatible with the presumed variability of the latency of childhood leukaemia among survivors of the atomic bombs (Finch, 1984) and the mobility of healthy children in the study population. These limits were set before examination of the data. However, other investigators in other sociocultural settings have reported clustering in smaller space–time intervals (Gunz and Spears, 1968; Gilman and Knox, 1995).

The expected number (E) of space–time pairs is obtained under the assumption that space proximity and time proximity are independent. Statistical evaluation of the observed (O) number of cases in the space–time cell is based on the Poisson distribution with mean equal to E (David and Barton, 1966); simulation studies conducted by ourselves and others have confirmed that the distribution of O under the null hypothesis is closely approximated by this. The corresponding *P*-value is one-tailed. In small values of E, *P*-values are calculated from the exact Poisson distribution (referred to subsequently as exact), but for larger E values ($>50$), the *P*-values are based on normal approximations. As a further aid to interpretation, a global test for space–time interaction with 15 time thresholds (1–15 months) and 15 space thresholds (0.5–7.5 km) has been applied (Bhopal *et al.*, 1992). Statistical testing has used Monte Carlo simulation with times of diagnosis allocated at random 499 times.

Evaluation of clustering was done for Greece as a whole as well as in urban, semi-urban and rural areas of the country as defined by the National Statistical Service of Greece (rural, less than 2000 inhabitants; semi-urban, 2000 to 9999; urban, 10 000 or more inhabitants). The primary analyses were for total leukaemia and acute lymphoblastic leukaemia in standard 5 year age groups for the whole country and for urban, semi-urban and rural areas. When these were completed, exploratory analyses were conducted for clustering *within* non-standard age groups (including ages 2–4 for ALL because of the prior hypothesis relating these cases to infections) and *between* standard 5 year age groups. For the latter, a simple modification of the Knox test was applied (Gilman and Knox, 1991).

### Results

Descriptive information concerning incidence of childhood leukaemia by type, gender, age and degree of urbanisation are shown in Table I. The descriptive pattern, including the early childhood peak, is similar to that in other population groups. The incidence rates in rural areas are lower than those in urban areas for each of the age groups 0–4 years

**Table I** Distribution of 872 cases of childhood leukaemia by type of leukaemia, age, gender, residence at diagnosis and incidence rates per 1 000 000 person–years (Greece, 1980–89)

| | | | Type of leukaemia | | | | | |
|---|---|---|---|---|---|---|---|---|
| | | | *Acute lymphoblastic* | | *Other type* | | *Total* | |
| *Area* | *Gender* | *Age* | *Number* | *Incidence* | *Number* | *Incidence* | *Number* | *Incidence* |
| Urban (10 000+) | Boys | 0–4 | 145 | 65.9 | 20 | 9.1 | 165 | 75.0 |
| | | 5–9 | 89 | 40.3 | 11 | 5.0 | 100 | 45.3 |
| | | 10–14 | 38 | 16.9 | 12 | 5.3 | 50 | 22.2 |
| | Girls | 0–4 | 118 | 56.5 | 19 | 9.1 | 137 | 65.6 |
| | | 5–9 | 71 | 33.9 | 15 | 7.2 | 86 | 41.1 |
| | | 10–14 | 25 | 11.7 | 10 | 4.7 | 35 | 16.4 |
| Semi-urban (2000–9999) | Boys | 0–4 | 22 | 45.3 | 5 | 10.3 | 27 | 55.6 |
| | | 5–9 | 27 | 55.2 | 2 | 4.1 | 29 | 59.3 |
| | | 10–14 | 10 | 19.7 | 4 | 7.9 | 14 | 27.6 |
| | Girls | 0–4 | 19 | 42.5 | 3 | 6.7 | 22 | 49.2 |
| | | 5–9 | 12 | 26.2 | 1 | 2.2 | 13 | 28.4 |
| | | 10–14 | 7 | 14.9 | 0 | 0.0 | 7 | 14.9 |
| Rural (<1999) | Boys | 0–4 | 43 | 42.2 | 2 | 2.0 | 45 | 44.2 |
| | | 5–9 | 20 | 18.9 | 6 | 5.7 | 26 | 24.6 |
| | | 10–14 | 19 | 17.0 | 10 | 9.0 | 29 | 26.0 |
| | Girls | 0–4 | 45 | 46.4 | 5 | 5.2 | 50 | 51.6 |
| | | 5–9 | 20 | 19.9 | 4 | 4.0 | 24 | 23.9 |
| | | 10–14 | 9 | 8.5 | 4 | 3.8 | 13 | 12.3 |
| Total | Boys | 0–4 | 210 | 56.7 | 27 | 7.3 | 237 | 64.0 |
| | | 5–9 | 136 | 36.2 | 19 | 5.1 | 155 | 41.3 |
| | | 10–14 | 67 | 17.3 | 26 | 6.7 | 93 | 24.0 |
| | Girls | 0–4 | 182 | 52.0 | 27 | 7.7 | 209 | 59.7 |
| | | 5–9 | 103 | 28.9 | 20 | 5.6 | 123 | 34.5 |
| | | 10–14 | 41 | 11.2 | 14 | 3.8 | 55 | 15.0 |

and 5–9 years, but the ALL childhood peak is evident in both urban and rural areas.

The results of applying Knox's test for evaluation of space–time clustering of all types of childhood leukaemia by level of urbanisation and age are presented in Table II. There is highly significant evidence for clustering of childhood (0–14 years) leukaemia in Greece as a whole, the observed number of pairs that are close in both space and time exceeding the expected number by 5.2% ($P$=0.004). This excess is mostly accounted for by the pattern in urban areas and it is particularly evident for leukaemia cases at ages 0–4 years, among whom the observed number of pairs that are close in both space and time exceeded the expected number by 9.4% ($P$=0.004). For the whole country or by level of urbanisation, there is no indication of space–time clustering for leukaemia cases older than 5 years.

The above findings for total childhood leukaemia also apply to the dominant subtype ALL (Table II). When analyses were restricted to ALL at 2–4 years of age in urban areas, the evidence for clustering was extremely strong (observed (O) space–time pairs=372, excess over expected (E) number=19%, $P$=0.0006) (Table III).

Although the *a priori* space–time limits were set at 5 km and one year, we have examined whether patterns were also discernible with smaller intervals. When 6 months instead of one year was used as the time limit with the space limit still at 5 km, the relative excess in Greece as a whole of childhood leukaemia at any age and type was more pronounced (O=1543, E=1434.20, $P$=0.002, excess=7.6%) as were the relative excess of childhood leukaemia of any type at age 0–4 years (O=455, E=408.12, $P$=0.01, excess=11.5%); the relative excess of ALL at any age (O=1093, E=1032.90, $P$=0.03, excess=5.8%); and the relative excess of ALL at age 0–4 years (O=334, E=314.24, $P$=0.14, excess=6.3%).

**Table II** Application of Knox's test for space–time clustering of childhood leukaemia cases in Greece 1980–89

| *Areas* | *All ages* | *0–4 years* | *5–9 years* | *10–14 years* |
|---|---|---|---|---|
| Childhood leukaemia of any type | | | | |
| Urban | | | | |
| Pairs | 163 878 | 45 451 | 17 205 | 3570 |
| n pairs within 5 km, 1 year | | | | |
| Observed | 2751 | 846 | 285 | 33 |
| Expected | 2640.0 | 776.7 | 283.3 | 41.2 |
| % Excess | 4.2% | 8.9% | 0.6% | deficit |
| *P*-value | 0.016 | 0.007 | 0.468 | – |
| Semi-urban | | | | |
| n pairs | 6216 | 1176 | 861 | 210 |
| n pairs within 5 km, 1 year | | | | |
| Observed | 8 | 2 | 1 | 1 |
| Expected | 7.6 | 1.5 | 1.4 | 0.2 |
| % Excess | 5.3% | 33.3% | ** | ** |
| *P*-value | 0.42 | 0.32 | ** | ** |
| Rural | | | | |
| n pairs | 17 391 | 4465 | 1225 | 861 |
| n pairs within 5 km, 1 year | | | | |
| Observed | 22 | 5 | 1 | 1 |
| Expected | 17.3 | 4.7 | 0.7 | 0.8 |
| % Excess | 27.2% | 6.4% | ** | ** |
| *P*-value | 0.13 | 0.42 | ** | ** |
| Total Greece | | | | |
| n pairs | 379 756 | 99 235 | 38 503 | 10 878 |
| Pairs within 5 km, 1 year | | | | |
| Observed | 2943 | 882 | 315 | 38 |
| Expected | 2798.8 | 806.1 | 303.2 | 45.9 |
| % excess | 5.2% | 9.4% | 3.9% | deficit |
| *P*-value | 0.004 | 0.004 | 0.256 | – |
| Acute lymphoblastic leukaemia in Greece (total) | | | | |
| n pairs | 272 691 | 76 636 | 28 441 | 5778 |
| Pairs within 5 km, 1 year | | | | |
| Observed | 2086 | 651 | 222 | 18 |
| Expected | 2007.7 | 619.9 | 213.1 | 24.5 |
| % excess | 3.9% | 5.0% | 4.2% | deficit |
| *P*-value | 0.04 | 0.11 | 0.28 | – |

**Not calulated when observed pairs=1.

**Table III** Additional analyses[a] of space–time clustering of acute lymphoblastic leukaemia cases in different age groups in urban and rural areas[b]

| *Age groups* | *Urban areas* | | | | *Rural areas* | | | |
|---|---|---|---|---|---|---|---|---|
| | *O* | *E* | *% excess* | P | *O* | *E* | *% excess* | P |
| Within 5–14 years | 363 | 361.30 | 0.47 | 0.47 | 5 | 2.01 | 149 | 0.05 |
| Between 0–4 and 5–9 years | 722 | 709.15 | 1.81 | 0.32 | 7 | 4.45 | 57 | 0.16 |
| Within 2–4 years | 372 | 312.97 | 18.9 | 0.0006 | 1 | 2.88 | deficit | – |
| Within 4–11 years | 930 | 914.12 | 1.74 | 0.30 | 10 | 5.15 | 94 | 0.04[c] |

[a]Data-driven analyses, apart from that concerning the 2–4 year age group. [b]For total leukaemia results are similar. [c]Nominal *P*-value with no adjustment for the large number of alternative age bands considered. O, observed; E, expected.

When the space limit was set at 2 km and the time limit at 6 months, the overall pattern was weaker and the smaller numbers of observed pairs hindered statistical substantiation. Thus, over Greece as a whole, for childhood leukaemia at any age and type O = 539, E = 523.08, $P$ = 0.25, excess 3.0%, for childhood leukaemia of any type at age 0–4 years O = 165, E = 142.94, $P$ = 0.04, excess = 15%; for ALL of any age O = 396, E = 388.56, $P$ = 0.36, excess = 1.9%; and for ALL at age 0–4 years O = 126, E = 115.96, $P$ = 0.19 excess = 8.7%. The global test confirmed the significance of the clustering for total childhood leukaemia and specifically for the age group 0–4 years (Monte Carlo $P$-value = 0.012). Substantial excess of observed over expected space–time pairs were seen for time thresholds of 3–15 months but were mainly associated with space thresholds of 4.5 km and over.

In rural areas, there are few observed and expected pairs of childhood leukaemia of any type close in both space and time but an intriguing pattern is discernible. Whereas observed and expected pairs that are close in both space and time are essentially equal when childhood leukaemia is studied in separate 5 year age groups, there is an excess of observed over expected pairs (27.2%) in the cluster cell when childhood leukaemia cases of all ages are examined together, although this is based on small numbers and is not statistically significant. Restricting space–time clustering in rural areas to ALL cases indicates that the phenomenon described for all leukaemia cases is almost wholly accounted for by cases of ALL. Thus, observed and expected pairs that are close in both space and time are essentially equal when ALL is studied in separate 5 year age groups, but these is an excess of observed over expected cases (18 *vs* 13.7, i.e. 31.4%) in the cluster cell when childhood ALL cases of all ages are examined together. Again, numbers are too small to allow statistical substantiation (exact $P$ = 0.13).

The inconsistency in rural areas between overall and age-specific results for childhood leukaemia in general and ALL in particular, must be attributable to space–time interaction *within* the 5–14 year age group or *between* the 0–4 year and older groups. Additional analyses were therefore performed, focusing on pairs of cases that belonged to different age groups. There was some evidence of clustering in rural areas within the 5–14 year age group, with all the space-time pairs being ALL, and between the 0–4 years and 5–9 years age groups, where for childhood leukaemia of any type O = 8, E = 4.90, $P$ = 0.12 and for ALL O = 7, E = 4.45, $P$ = 0.16. Further analyses identified the age range 4–11 years as having stronger evidence of clustering in rural areas ($P$ = 0.04; Table III). There was no evidence of any of these phenomena in urban areas or of clustering involving the 2–4 years group in rural cases (Table III). Although the results for rural areas are based on small numbers, they suggest that clustering in these areas involves cases over a broader and older age range than in the urban areas. This would be consistent with a delay in the establishment of immunity against a putative agent for childhood leukaemia among very young children.

Establishment of herd immunity is delayed in rural areas, allowing the occasional excess occurrence of the corresponding infectious diseases among older children in this population. It is of interest that when space–time limits were set at 2 km and 6 months, clustering of ALL cases within the 5–14 year age group was very pronounced and statistically highly significant (O = 5, E = 1.01, $P$ = 0.004); however, these limits had not been specified *a priori*.

Since space–time clustering overall was noted only among childhood leukaemia at 0–4 years ($N$ = 446), we have tried to determine whether the tendency for space–time clustering was stronger for particular case subgroups defined in terms of gender, age and leukaemia type. Among the 446 cases at 0–4 years, 180 were not involved in a clustering pair; 112 were involved in 1–3 clustering pairs; 79 were involved in 4–10 clustering pairs; and 75 in more than 10 clustering pairs. There was no evidence that age (in exact years) or gender of the index case had any influence on the clustering pattern. Among the 180 non-clustering cases, 156 (87%) were ALL and among the 266 clustering cases 237 (89%) were ALL. Of some interest is that ALL was more frequently the diagnosis when the index case was involved in relatively few (1–10) clustering pairs (175 out of 191, or 92%) than when the index case was involved in many (more than 10) clustering pairs (61 out of 75, or 82%).

### Discussion

In the absence of a candidate virus or other agent, the search for an infectious aetiology for childhood leukaemia has attempted to ascertain whether the pattern of occurrence of the disease resembles that of other diseases caused by agents of moderate infectivity but low pathogenicity; these diseases usually result in apparently separate cases emerging from a pool of infected transient carriers, immune persons and uninfected individuals. Cases of polio before the vaccination era and cases of meningococcal meningitis follow this pattern of occurrence. Various authors have used alternative approaches in their efforts to characterise the parameters of childhood leukaemia epidemicity, if any, and establish the likelihood of an underlying infectious process. Space–time clustering was the first formal statistical methodology to investigate clustering and remains the method of choice if latency is short. Even when temporal clustering alone or spatial clustering alone are not demonstrable, space–time clustering is a common characteristic of epidemics of infectious origin, provided that the limits defining the space–time clusters accommodate basic parameters of the infectious process, including infectivity, period of communicability, susceptibility, pathogenicity and latency. It is not clear whether these conditions apply with respect to childhood leukaemia.

Knox's method represents an ingenious approach to assess space–time clustering or the detection of interrelated cases that are caused (at least in part) by an agent that changes position in space with the passage of time. This method is highly dependent on the space and time limits used for the evaluation of the clustering (Knox, 1964*a*). Several investigations have assessed space–time clustering of childhood leukaemia. These studies have been reviewed by Linet (1985) and Alexander (1993) both of whom felt that the consensus provided some support for space–time clustering. The evidence, however, is far from conclusive. One problem is intrinsic to the situation under investigation: the likelihood that patterns will be unpredictable, since even agents with high pathogenicity leading to a high proportion of clinically overt cases can follow unpredictable patterns when the period of communicability and disease latency are prolonged or variable. The second problem is statistical and reflects the interpretation of test statistics when space and/or time limits are data-derived or chosen as part of a multiple testing process.

The present study has used the results of previous investigations which have suggested the space (= 5 km) and time (= 1 year) limits as biologically meaningful and empirically efficient grid references for the detection of space–time clustering in childhood leukaemia (Meighan and Knox, 1965; Mainwaring, 1966; Smith *et al.*, 1976; Morris, 1990). Certain conditions prevailing in Greece, including population movements from rural and smaller urban communities producing substantial mixing in growing urban centres, in contrast to the isolation of mountainous villages, may also represent strengths of the present study by creating conditions for small epidemics (Kinlen and Petridou, 1995). Among the weakness of the present study are: (1) the possible underascertainment of cases of childhood leukaemia since a cancer registry was not in operation at the time these cases were ascertained. However, general underascertainment cannot compromise the validity of Knox's method for the detection of clustering (Knox, 1964*a*), since expected values are conditional on the margins; (2) the multiplicity of the physicians involved in the diagnosis of cases of childhood leukaemia and the unfortunate absence of immunophenotyping for all but a small proportion of the cases implies some amount of misclassification between the various leukaemia types. However, the specificity on the ascertainment of leukaemia itself is certainly very high, given the clinical manifestation and the natural history of the disease.

Several findings of the present study support the hypothesis that an infectious agent is involved in the aetiology of childhood leukaemia: (1) There was an overall space–time clustering that was statistically significant in the country as a whole and in the urban areas; the clustering was evident among cases aged 0–4 years in agreement with most of the earlier reports reviewed by Linet (1985) and Alexander (1993). The concentration of clustering in the urban areas may simply be due to the much larger number of pairs in these areas (Table II). Alternatively, it may reflect an unusual instability in the Greek urban population's exposure to the elusive relevant infection–subsequent to intense internal migration into towns and extensive population mixing (Kinlen and Petridou, 1995). (2) Among children aged 0–4 years clustering was concentrated in ALL and particularly in the childhood peak age range (2–4 years) of ALL which has been the focus of infectious aetiology hypotheses. (3) In rural areas there was a suggesting of clustering of cases belonging to broader and older age groups implying that a later development of herd immunity allowed the effective infection of older children in spite of the lower overall infection density in the rural population. An intriguing finding was that cases of ALL were frequently involved in clusters with a limited number of pairs (1–10) but were less frequently involved in clusters with many pairs (more than 10) and were also less frequent among cases forming no pairs at all. The latter result is compatible with the postulated infectious origin of ALL but the former could mean either that the whole pattern is due to chance or that clusters with many pairs reflect a methodological artefact or a distinct aetiological process of possibly fixed environmental origin of varying time intensity.

Accumulating recent evidence of space–time clustering of childhood leukaemia (Draper, 1991), findings from studies probing the effects on leukaemia rates of population mixing (Kinlen *et al.*, 1990, 1991; Kinlen and Hudson, 1991; Kinlen and Petridou, 1995), data from studies assessing the effect of early day care attendance (Van Steensal-Moll *et al.*, 1986; Petridou *et al.*, 1993), and biological considerations (Caldwell, 1983, Greaves, 1988; Alexander, 1993; Greaves and Alexander, 1993) strengthen the evidence that a common infection of high virulence but of low pathogenicity may be involved in the aetiology of childhood leukaemia. The evidence is not entirely consistent but no alternative causal interpretation better accommodates the overall evidence. The present study shifts the centre of gravity of this evidence one step closer to the hypothesis invoking an infectious agent in the multifactorial aetiology of childhood leukaemia.

**Acknowledgements**
This study was supported in part by the Europe Against Cancer Program.

### References

ALEXANDER FE. (1993). Viruses, clusters and clustering of childhood leukemia: a new perspective? *Eur. J. Cancer*, **29**, 1424–1443.

ALEXANDER FE, RICKETTS TJ, MCKINNEY P AND CARTWRIGHT RA. (1990). Community lifestyle characteristics and risk of acute lymphoblastic leukemia in children. *Lancet*, **336**, 1461–1464.

BHOPAL RS, DIGGLE P AND ROWLINGSON B. (1992). Pinpointing clusters of apparently sporadic cases of Legionaires' disease. *Br. Med. J.*, **304**, 1022–26.

CALDWELL CG. (1983). Infections, infestations and cancer. In *The Epidemiology of Cancer*. Bourks GJ (ed.) pp. 292–326. Charles Press: Philadelphia.

DAVID FN AND BARTON DE. (1966). Two space–time interaction tests for epidemicity. *Brit. J. Prev. Soc. Med.*, **20**, 44–48.

DAVIS JG, BENNETT WR, BRADY JV, BRENT RL, GORDIS L, GORDON WE, GREENHOUSE SW, REITER JR, STEIN GS, SUSSKIND CH AND TRICHOPOULOS D. (1992). Health effects of low-frequency electric and magnetic fields. Report of an Oak Ridge Associated Universities Panel for the Committee on Interagency Radiation Research and Policy Coordination. ORAU 92/F8, pp. xiv, 372. Oak Ridge Associated Universities: Washington DC.

DOLL R. (1989). The epidemiology of childhood leukemia. *J. R. Stat. Soc.*, (Series A), **152**, 341–351.

DRAPER G (ed.). (1991). The geographical epidemiology of childhood leukemia and non-Hodgkin lymphomas in Great Britain, 1966–1983. HMSO: London.

ELLERMAN V AND BANG O. (1908). Experimentalle leukemia bei huhnern. *Centre Bakteriol. Abt. I Orig.*, **46**, 595–609.

FINCH SC. (1984). Leukemia and lymphoma in atomic bomb survivors. In *Radiation Carcinogenesis: Epidemiology and Biological Significance*, Boice JD, Fraumeni JF (eds.) pp. 37–44. Raven Press: New York.

GEOGRAPHICAL INFORMATION SYSTEM. (1987). MAPINFO CO. Park: Troy NY.

GILMAN EA AND KNOX EG. (1991). Temporal–spatial distribution of childhood leukemias and non-Hodgkin lymphomas in Great Britain. In *The Geographical Epidemiology of Childhood Leukemia and non-Hodgkin Lymphomas in Great Britain*, 1966–83. Draper G (ed.). pp. 77–100. HMSO: London.

GILMAN EA AND KNOX EG. (1995). Childhood cancers: space–time distribution in Britain. *J. Epidemiol. Community Health*, **49**, 158–163.

GREAVES MF. (1988). Speculations on the cause of childhood acute leukemia. *Leukemia*, **2**, 120–125.

GREAVES MF AND ALEXANDER FE. (1993). An infectious aetiology for childhood leukemia? *Leukemia*, **7**, 349–360.

GROSS L. (1978). Viral origin of cancer and leukemia: a look into the past, present and future. GHA Clowes Memorial Lecture. *Cancer Res.*, **38**, 485–493.

GROVES FD, LINET MS AND DEVESA SS. (1995). Patterns of occurrence of the leukemias. *Eur. J. Cancer*, **31**, 941–949.

GUNZ FW AND SPEARS GFS. (1968). Distribution of acute leukemia in time and space. Studies in New Zealand. *Br. Med. J.*, **4**, 604–608.

KASSIMOS D. (1992). *The Epidemiology of Childhood Leukemia in Greece*. Doctoral dissertation. Athens University Medical School, Athens, Greece.

KINLEN LJ AND HUDSON C. (1991). Childhood leukemia and poliomyelitis in relation to military encampments in England and Wales in the period of national military service, 1950–63. *Br. Med. J.*, **303**, 1357–1362.

KINLEN LJ AND PETRIDOU E. (1995). Childhood leukemia and rural population movements in Greece, Italy and other countries. *Cancer Causes Control*, **6**, 445–450.

KINLEN L, CLARKE K AND HUDSON C. (1990). Evidence from population mixing in British new towns 1946–1985 of an infective basis for childhood leukemia. *Lancet*, **336**, 577–582.

KINLEN LJ, HUDSON C AND STILLER LA. (1991). Contacts between adults as evidence for an infective origin of childhood leukemia: an explanation for the excess near nuclear establishments in West Berkshire? *Br. J. Cancer*, **64**, 549–554.

KNOX G. (1963). Detection of low intensity epidemicity. Application to cleft lip and palate. *Br. J. Prev. Soc. Med.*, **17**, 121–127.

KNOX G. (1964*a*). The detection of space–time interactions. *Appl. Stat.*, **13**, 25–29.

KNOX G. (1964*b*). Epidemiology of childhood leukemia in Northumberland and Durham. *Br. J. Prev. Soc. Med.*, **18**, 17–24.

KNOX EG. (1994). Leukemia clusters in childhood: geographical analysis in Britain. *J. Epidemiol. Community Health*, **48**, 369–376.

LENOIR GM, O'CONOR GT AND OLWENY CLM (eds.). (1985). Burkitts' Lymphoma: a Human Cancer Model. WHO/IARC: Lyon.

LINET MS. (1985). *The Leukemias: Epidemiological Aspects*. Monographs in Epidemiology and Biostatistics. Oxford University Press: New York.

MACMAHON B. (1992). Is acute lymphoblastic leukemia in children virus-related? *Am. J. Epidemiol.*, **136,** 916–924.

MAINWARING D. (1966). Epidemiology of acute leukemia in Liverpool area. *Br. J. Prev. Soc. Med.*, **20,** 189–194.

MEIGHAN SS AND KNOX G. (1965). Leukemia in childhood. Epidemiology in Oregon. *Cancer*, **18,** 811–814.

MORRIS V. (1990). Space–time interactions in childhood cancer. *J. Epidemiol. Community Health*, **44,** 55–58.

MUELLER N. (1991). The epidemiology of HTLV-I infection. *Cancer Causes Control*, **2,** 37–52.

PETRIDOU E, HSIEH C-C, KOTSIFAKIS G, SKALKIDIS Y, FLYTZANI V AND TRICHOPOULOS D. (1992). Evolution and distribution of childhood leukemia in Greece: Etiological implications. *Eur. J. Public Health*, **2,** 29–33.

PETRIDOU E, KASSIMOS D, KALMANTI M, KOSMIDIS H, HAIDAS S, FLYTZANI V, TONG D AND TRICHOPOULOS D. (1993). Age of exposure to infections and risk of childhood leukemia. *Br. Med. J.*, **307,** 774.

PETRIDOU E, PROUKAKIS CH, TONG D, KASSIMOS D, ATHANASIADOU-PIPEROPOULOU F, HAIDAS S, KALMANTI M, KOLIOUSKAS D, KOSMIDIS H, LOUIZI A, SIMOPOULOS S AND TRICHOPOULOS D. (1994*a*). Trends and geographical distribution of childhood leukemia in Greece in relation to the Chernobyl accident. *Scand. J. Soc. Med.*, **22,** 127–131.

PETRIDOU E, KOSMIDIS H, HAIDAS S, TONG D, REVINTHI K, FLYTZANI V, PAPAIOANNOU D AND TRICHOPOULOS D. (1994*b*). Survival from childhood leukemia depending on socioeconomic status in Athens. *Oncology*, **51,** 391–395.

SMITH PG, PIKE MC, TILL MM AND HARDISTY RM. (1976). Epidemiology of childhood leukemia in Greater London: a search for evidence of transmission assuming a possibly long latent period. *Br. J. Cancer*, **33,** 1–8.

VAN STEENSAL-MOLL HA, VALKENBURG HA AND VAN ZANEN GE. (1986). Childhood leukemia and infectious diseases in the first year of life: a register based case–control study. *Am. J. Epidemiol.*, **124,** 590–594.